# Structure Elucidation of a Polysaccharide from *Umbilicaria esculenta* and Its Immunostimulatory Activity

**DOI:** 10.1371/journal.pone.0168472

**Published:** 2016-12-20

**Authors:** Bi-Wei Zhang, Jin-Long Xu, Hua Zhang, Qiang Zhang, Jie Lu, Jun-Hui Wang

**Affiliations:** 1 School of Biotechnology and Food Engineering, Hefei University of Technology, Hefei, People’s Republic of China; 2 Anhui Qiangwang Flavouring Food Co., LTD, Dongcheng Development Zone, Jieshou City, Anhui, China; University of Insubria, ITALY

## Abstract

*Umbilicaria esculenta* has been used as a tonic food in China for several centuries owing to its pleasant flavor and health benefits. In this study, a water soluble polysaccharide, which we designated as UP2, with an average molecular weight of 3.33 × 10^5^ Da, was isolated from *U*. *esculenta* cultivated in the Huangshan Mountain, by consecutive hot water extraction and anion-exchange chromatography. Gas chromatography analysis indicated that UP2 contained three kinds of monosaccharides, including mannose, glucose, and galactose at a molar ratio of 1.7:1.0:1.2. Linkage analysis of UP2 revealed the presence of (1 → 6)-linked glucosyl, (1 → 3,6)-linked glucosyl, *t*-linked galactosyl, (1 → 6)-linked galactosyl and (1 → 6)-linked mannosyl at a molar ratio of 0.7:4.6:4.1:2.2:9.1. Structural analysis determined that UP2 possessed a backbone consisting of (1 → 6)-linked *β-D*-glucopyranosyl and (1 → 6)-linked *α*-*D*-mannopyranosyl residues, which substituted at the *O*-3 position of (1 → 6)-linked *β-D*-glucopyranosyl residues by branches of (1 → 6)-linked *α*-*D*-galactopyranosyl and 1-linked *β*-*D*-galactopyranosyl residues. Immunostimulatory activity analysis showed that UP2 could stimulate the proliferation of RAW264.7 cells in a dose-dependent manner, and all the samples (20–500 μg/mL) were found to enhance nitric oxide production. The highest phagocytic activity of UP2 was observed at 200 μg/mL. Thus, UP2 may be a potential source of biological and pharmacological agents.

## Introduction

Polysaccharides are found in animal cell membranes and cell walls of plants and microorganisms [1–5], and are compound of saccharide monomers linked through glycosidic bonds formed through their aldehyde and keto groups [6]. They are essential biological macromolecules, and are directly involved in life processes, exhibiting a variety of important biological functions [2, 7]. Furthemore, polysaccharides exhibit immune-enhancement, anti-tumor, antioxidant, and other biological activities [8–9]. Consequently, much research attention has been focused on the extraction and purification, structural elucidation, biological and pharmacological effects, and structure-activity relationships of polysaccharides. Most of polysaccharides are comparatively nontoxic immunomodulatory agents [4, 10]. The biological activity of a polysaccharide is closely related to its physicochemical properties, such as its total sugar content, molecular weight, and glycosidic linkages [3, 11–13]. It has been demonstrated that the immunological activity of polysaccharides manifested through the activation of T cells, B cells, natural killer cells, and the complement system to stimulate activation of macrophages to produce cytokines and exert immunity modulating activity [14–15].

Lichens are widely-distributed symbiotic organisms that consist of a fungus and an algae (the photobiont) [16–17] and ca. 13,500 distinct species of lichens have been identified [9]. In recent years, polysaccharides isolated from lichens have been shown to exert antitumor, antioxidant, antiviral, immunomodulation, and other biological effects [18–19], indicating that polysaccharides derived from lichens may be applied in pharmacology and the food industry. It was reported that polysaccharide from *Umbilicaria proboscidea*, with (1→6)-linked *β*-glucan backbone, had the potential to induce anti-inflammatory effects [20]. The polysaccharides isolated from the lichens of *umbilicaria* species, consisting mainly of (1→6)-*β*-glucan, showed a remarkable anti-tumor effect [21]. Therefore, research into polysaccharides derived from lichens is an important ongoing concern.

*Umbilicaria esculenta* is a precious edible and medicinal lichen, and has been employed in traditional Chinese medicine for several centuries [1] to treat inflammation, bleeding, and poisoning. The polysaccharide components of *U*. *esculenta* have been demonstrated to exhibit anti-tumor, anti-HIV [21–22], and antithrombotic activities [9, 23]. It is widely known that the chemical structure of polysaccharides has a profound effect on their bioactivities [24]. However, little information is available on the structures and biochemical mechanisms of polysaccharides from *U*. *esculenta*.

We have previously reported the extraction and preliminary characterization of polysaccharides from *U*. *esculenta* cultivated in the Huangshan Mountain, and demonstrated that the crude polysaccharides exhibited immunomodulatory activities *in vitro* [1, 25]. However, those studies were not concerned with the detailed chemical structures or mechanisms of immunomodulatory activity exhibited by the polysaccharides from *U*. *esculenta*, and nor were their structure-activity relationships fully investigated. In addition, the results of preliminary experiments indicated that UP2 showed a remarkable biological effect and prompted us to investigate the immunostimulatory activity of UP2. Therefore, we herein present a detailed analysis of the chemical structures, physical properties, and mechanisms of immunomodulatory activity of the polysaccharides from *U*. *esculenta*.

## Materials and Methods

### Materials and reagents

*Umbilicaria esculenta* were purchased from Huangshan green meetall the organic food development Co., Ltd. (Huangshan, China). The production license number is QS3410 1601 0291. The batch number is 20120907. Diethylaminoethyl (DEAE) cellulose, standard monosaccharides (D-glucose, D-mannose, D-xylose, L-galactose, L-rhamnose, and L-arabinose), T-series dextrans (molecular weight 1.0, 2.0, 15.0, 40.0, 50.0, and 200.0 KDa), trifluoroacetic acid (TFA) and dimethyl sulfoxide (DMSO) were purchased from Sigma–Aldrich Co., Ltd. (St. Louis, MO, USA). 3-(4,5-Dimethylthiazol-2-yl)-2,5-diphenyltetrazolium bromide (MTT), lipopolysaccharide (LPS), penicillin-streptomycin solution, and Dulbecco's modified Eagle's medium (DMEM) were purchased from Hyclone Company. Newborn calf serum was purchased from Sijiqing Co., Ltd. (Hangzhou, China). All other reagents of analytical grade and distilled water were used for all reagent solutions.

### Isolation and purification of polysaccharides

The thallus of *U*. *esculenta* (100 g) was pulverized into powder, then defatted and decolorized in a Soxhlet extractor with acetone and ethyl acetate, respectively, for 48h each [26]. The defatted powder was then extracted twice with hot distilled water (1:30, *w/v*) at 100°C for 2 h. The extracted solution was filtered through four pieces of gauzes to obtain the supernatants [8]. After filtration, the supernatants were combined and concentrated. Subsequently, the resultant solution was deproteinized using the Sevag method. The resulting aqueous solution was decolorized with 30% Hydrogen Peroxide (H_2_O_2_) and dialyzed against water (molecular weight cut off 3500 Da) for seven days. The dialysate was centrifuged to remove insoluble matter, concentrated, and lyophilized to give the crude polysaccharide, hereafter designated UP.

UP (100 mg) was dissolved in distilled water (10 mL) and fractionated on a DEAE cellulose chromatography column (3.0 × 40 cm), eluted with distilled water, 0.1 M Sodium chloride (NaCl), and 0.3 M NaCl in turn at a flow rate of 2.5 mL/min. The eluate was collected by an automatic collector (10 mL/tube) and monitored using the phenol-sulfuric acid method. Each eluted fraction was concentrated and dialyzed. The corresponding dialyzsate was concentrated and lyophilized to obtain three polysaccharide fractions, hereafter termed UP1 (eluted with distilled water), UP2 (eluted with 0.1 M NaCl) and UP3 (eluted with 0.3 M NaCl). The homogeneous UP2 fraction was subjected to subsequent analyses.

### Homogeneity and molecular weight

The homogeneity and molecular weight of UP2 were measured using high-performance gel permeation chromatography (HPGPC) [8] on a Waters E2695 high performance liquid chromatography (HPLC) system apparatus equipped with a Ultrahydrogel^TM^ 2000 column (7.8 mm × 300 mm), a Ultrahydrogel^TM^ 500 column (7.8 mm × 300 mm), and a Waters 2424 evaporative light scattering detector (Waters Corporation, USA). The sample was dissolved in ultrapure water at a concentration of 1.0 mg/mL and passed through a 0.22 μm filtration membrane. A 20 μL aliquot of the sample solution was injected with ultrapure water as the eluent at a flow rate of 0.5 mL/min. A calibration curve for molecular weight measurement with retention time plotted against the logarithm of the molecular weight was constructed using a set of dextran standards (T-10, T-20, T-150, T-400, T-500, T-2,000).

### Determination of the total sugar, uronic acid and protein contents

The total sugar content was measured by the phenol-sulfuric acid method [27–28] using glucose as the standard at 490 nm. The protein content was determined using the Coomassie Brilliant Blue G-250 method [29] with bovine serum albumin as the standard at 595 nm. The uronic acid content of UP2 was analyzed using the *m*-hydroxylbiphenyl method [30] with galacturonan as the standard at 520 nm.

### UV-visible (UV-Vis) and Fourier-transform infrared (FT-IR) spectroscopic analysis

The UV Vis absorption spectrum of UP2 was obtained on a UV-visible spectrophotometer (Beijing Purkinje General Instrument Co., Ltd, China) in the spectral scan range of 900–190 nm^-1^ at room temperature.

FT-IR spectroscopy was performed on a Nicolet 5700 FT-IR spectrometer (Thermo Nicolet, USA) using the KBr disc method [31] at room temperature in the wavelength range of 4000–400 cm^-1^.

### Monosaccharide composition analysis

Determination of the monosaccharide composition of UP2 was performed using gas chromatography (GC). UP2 (5.0 mg) was hydrolyzed with 2M TFA (4.0 mL) in a sealed tube at 120°C for 4 h. After removing the residual TFA with a rotary evaporator, the hydrolysate was dissolved in ultrapure water (3.0 mL) and reduced with Sodium borohydride (NaBH_4_) (30 mg) at room temperature for 3 h. The resultant solution was neutralized with 25% acetic acid, and then evaporated to dryness in a rotary evaporator. Then, 3.0 mL of each acetic anhydride and pyridine were added, and the reaction was sealed and reacted at 100°C for 1 h. The resulting monosaccharides were converted into alditol acetate derivatives [8, 32] and analyzed by GC.

### Methylation analysis

UP2 (35 mg) was dried in a vacuum oven for 3 h and dissolved in dried DMSO (5 mL) in a 50 mL three-necked flask. SMSM (2.0 mL) was added rapidly under nitrogen, and the mixture was stirred at room temperature for 0.5 h. Then 2.0 mL of methyl iodide was added dropwise to the mixed solution in an ice-salt bath under dark conditions. After ca. 1 h, the mixture was stirred in an oil bath at 50°C for 1 h, and then overnight at room temperature. Finally, the reaction solution was dialyzed and lyophilized to afford the methylated polysaccharide. Complete methylation was confirmed by the disappearance of the OH band (3200–3700cm^-1^) from the IR spectrum. The methylated polysaccharide was then hydrolyzed, reduced, acetylated, and converted into methylated alditol acetates [8, 32–34]. The resulting alditol acetates were analyzed by gas chromatography-mass spectrometry (GC-MS).

### NMR spectroscopy

1D and 2D NMR spectra were recorded on a VNMRS600 NMR spectrometer (Agilent). UP2 (60 mg) was dried in a vacuum oven over P_2_O_5_ at 45°C for 48 h, then dissolved in 99.9% D_2_O (1.0 mL) and transferred to a 5 mm NMR tube.

### Partial acid hydrolysis

UP2 (50 mg) was partially hydrolyzed with 8 mL of 0.5 M TFA at 100°C for 1 h. The TFA was subsequently removed on a rotary evaporator. The hydrolysate was dissolved in distilled water and dialyzed against distilled water for 48 h in a dialysis bag (molecular weight cut off 3500 Da) to obtain two fractions. The fraction inside the dialysis bag was concentrated and lyophilized to obtain a fraction referred to hereafter as UP2-0.5M, which was further analyzed by GC.

### Cell culture

Murine macrophage RAW264.7 cells were supplied by Professor Jian Liu (Hefei University of Technology, Hefei, China). The cells were maintained at 37°C in DMEM medium supplemented with 10% (*v/v*) newborn calf serum, 100 IU/mL penicillin, and 100 μg/mL streptomycin in an incubator with a humidified 5% CO_2_ atmosphere.

### Proliferation assay

The assay for murine macrophage cell line RAW264.7 proliferation was determined according to the MTT method [1, 26]. In brief, the logarithmic growth phase of RAW264.7 cells (150μL/well) was seeded into a 96-well plate at a density of 1.0 × 10^5^ cells/mL in DMEM medium. The cells were allowed to adhere for 24 h. UP2 (50 μL) at different concentrations (20, 50, 100, 200, and 500 μg/mL) was added to the wells, giving a final volume of 200 μL. LPS (50 μL, 10 μg/mL) was used as a positive control, and an equal volume of PBS buffer was used as a blank. Each group was repeated in six wells. The 96-well plate was placed in the incubator at 37°C with a humidified 5% CO_2_ atmosphere. After 48 h incubation, MTT solution (20 μL, 5 mg/mL) was added to each well, and the plate was further cultured for 4 h. After centrifugation at 1000 rpm for 5 min at 4°C, the supernatant was discarded, 100 μL of DMSO was added to each well, and the plate was shaken for 10 min at room temperature. Murine macrophage cell line RAW264.7 proliferation was recorded by the optical density at 570 nm using a multifunctional microplate reader (Multiskan Go 1510, Thermo Fisher Scientific). The proliferation index (PI) was calculated by the following formula:

$$PI=\frac{A_{1}}{A_{0}}$$

where A_0_is the absorbance of the control (without sample) and A_1_ is the absorbance of the sample.

### Determination of Nitric oxide (NO)

To determine the levels of NO in the RAW264.7 cells, NO content was estimated using the Griess reaction [1, 3, 15]. The logarithmic growth phase of RAW264.7 cells (150 μL/well) was seeded into 96-well plates at a density of 1.0 × 10^5^ cells/mL in DMEM medium, and incubated for 24 h in an incubator with a humidified 5% CO_2_ atmosphere at 37°C. Then, UP2 (50 μL) at different concentrations (20, 50, 100, 200, and 500 μg/mL) was added to the wells. LPS at a concentration of 10 μg/mL was used as a positive control and an equal volume of PBS buffer was used as a blank. Each group was repeated in 6 wells. The 96-well plate was placed in an incubator with a humidified 5% CO_2_ atmosphere at 37°C. After 48h stimulation, the cell supernatant (100 μl) and an equal volume of Griess reagent (1% sulfanilamide in 5% phosphoric acid and 0.1% N-[1-naphthyl]-ethylenediamine dihydrochloride in distilled water) were mixed in 96-well plates and incubated at room temperature with gentle shaking for 10 min. The absorbance was measured at 540 nm using a multifunctional microplate reader using Sodium nitrite (NaNO_2_) as a standard. A calibration curve was created using standard NaNO_2_ solutions with Griess treatment.

### Phagocytosis assay

A phagocytosis assay with murine macrophage cell line RAW264.7 was performed using the neutral red method [1, 3]. The logarithmic growth phase of RAW264.7 cells (150 μL/well) was seeded into 96-well plates at a density of 1.0 × 10^5^ cells/mL in DMEM medium and incubated for 24 h in an incubator with a humidified of 5% CO_2_ atmosphere at 37°C. Then, UP2 (50 μL) at different concentrations (20, 50, 100, 200, and 500 μg/mL) was added to the wells. LPS at a concentration of 10 μg/mL was used as a positive control and an equal volume of PBS buffer was used as a blank. Each group was repeated in six wells. The 96-well plate was placed in an incubator with a humidified of 5% CO_2_ atmosphere at 37°C. After 48 h incubation, the supernatant was discarded and the adhered cells were washed with PBS buffer twice. Then 100 μL of neutral red solution (0.1%, *w/v*) was added to each well, and the plate was cultured for 4 h. After incubation, the supernatant was removed and the adhered cells were washed twice with PBS buffer to remove the residual neutral red solution. Then, 100 μL of cell lysate (ethanol and acetic acid at a ratio of 1:1) was added to each well, and the plate was kept at room temperature for 2 h. Finally, the absorbances were measured at 540 nm using a multifunctional microplate reader.

### Statistical analysis

All experiments were repeated at least three times. The data values are expressed as mean ± SD (n ≥ 3). Statistical analysis was performed by the Single-factor ANOVA test in Statistical Analysis software SPSS 22.0. Significance was defined as a *P* value of < 0.05.

## Results and Discussion

### Isolation and purification analysis of UP2

**Fig 1:**
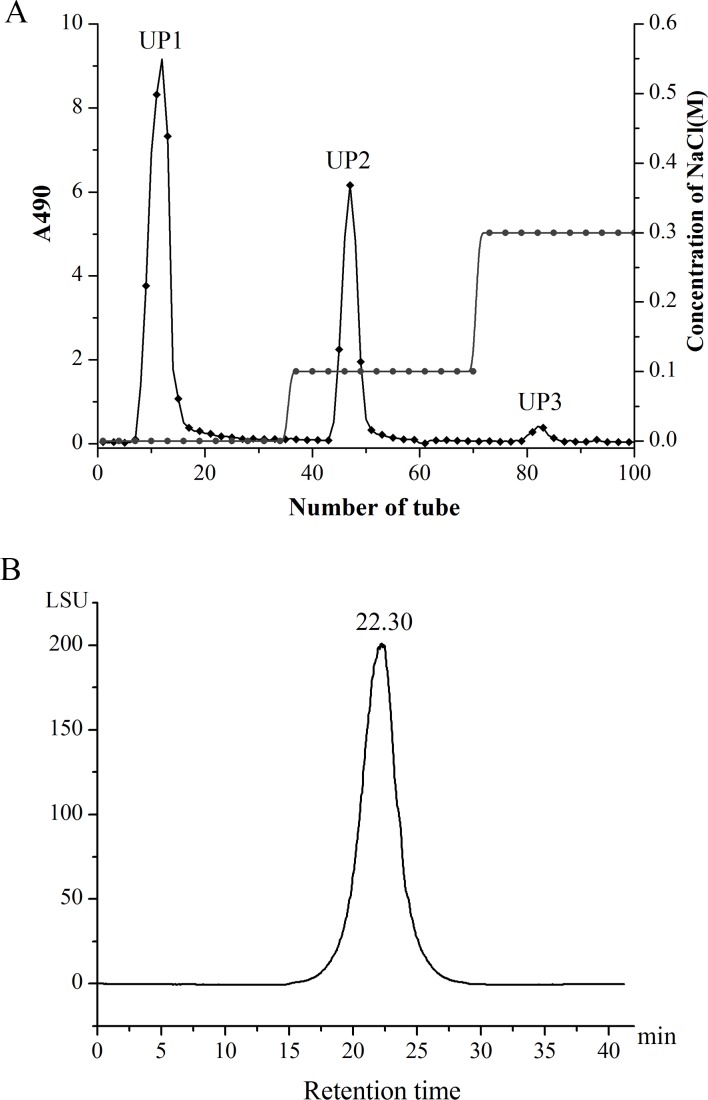
(A) anion-exchange chromatography elution profiles of crude UP on a column of DEAE-cellulose; (B) HPGPC chromatogram of UP2.

The water-soluble polysaccharide UP (5.68 g) was obtained from the thallus of *U*. *esculenta* cultivated in the Huangshan Mountain using the hot water extraction method. It was further purified by anion-exchange chromatography on a column of DEAE-cellulose to afford three fractions (**Fig 1A**), i.e., UP1 (eluted with distilled water), UP2 (eluted with 0.1M NaCl), and UP3 (eluted with 0.3M NaCl). All subsequent analysis focused on UP2. UP2 exhibited a single and symmetrical peak in its high-performance liquid chromatography (HPLC) chromatogram (**Fig 1B**), indicating that it is a homogeneous polysaccharide. Its molecular weight was ca. 3.33 × 10^5^ Da based on reference to dextran standards. The minimal and the maximum MW of UP2 were estimated to be 2.46 ×10^3^ Da and 4.06 ×10^7^ Da, respectively. The average molecular weight of tiger lily polysaccharide, probably identical to UP2, was estimated to be 3.51 × 10^5^ Da [35]. Two galactofuranomannans were isolated from the *Thamnolia vermicularis* var. *subuliformis*, their average molecular weights were estimated to be 1.90 × 10^4^ and 2.00 × 10^5^ Da, respectively [36]. Chemical analysis indicated that the total sugar content of UP2 was 97.65% (*w/w*), and its uronic acid content was below the detection limit. A negative response in Coomassie BBrilliant Blue G-250 analysis [29] and the lack of absorption at 260 or 280 nm in the UV spectra (data not shown) indicated that polysaccharide UP2 did not contain proteins or nucleic acids.

### FT-IR analysis of UP2

**Fig 2:**
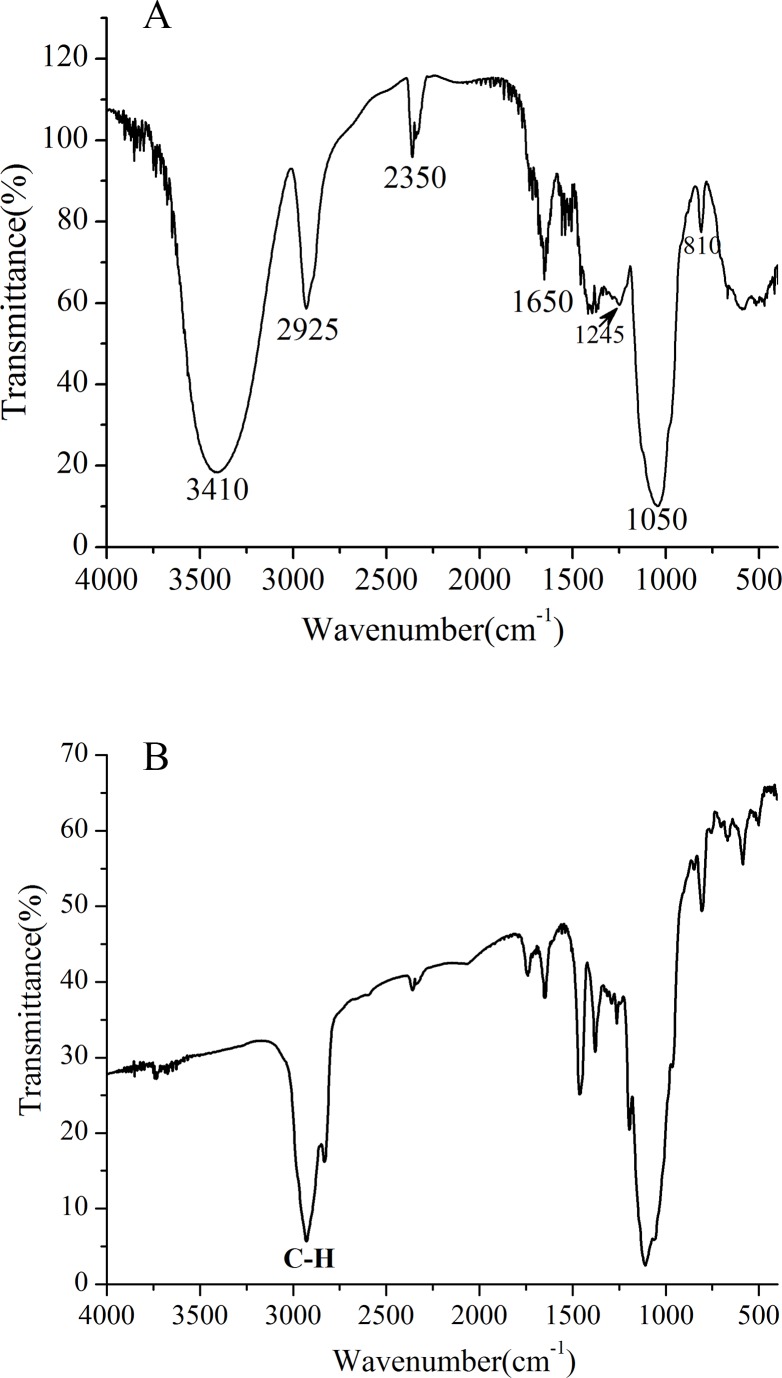
(A) FT-IR spectrum of UP2; (B) FT-IR spectrum of the methylated polysaccharide.

The FT-IR spectrum of UP2 was presented in **Fig 2A**, and showed the distinct characteristic absorption peaks of polysaccharides. The strong and broad peak at 3410 cm^-1^ was mainly due to O-H stretching vibration. The bands in the region of 2925 cm^-1^ and 1650 cm^-1^ were assigned to C-H stretching vibrations and associated water [4], respectively. In addition, the weak peak at 1245 cm^-1^ was attributed to deformation vibrations of the C-H bond [10]. The strong band around 1050 cm^-1^ was ascribed to the pyranose ring [6, 37]. The mannan content of the sample presented its typical absorption peaks at 810cm^-1^ [11].

### Structural characterization of UP2

**Fig 3:**
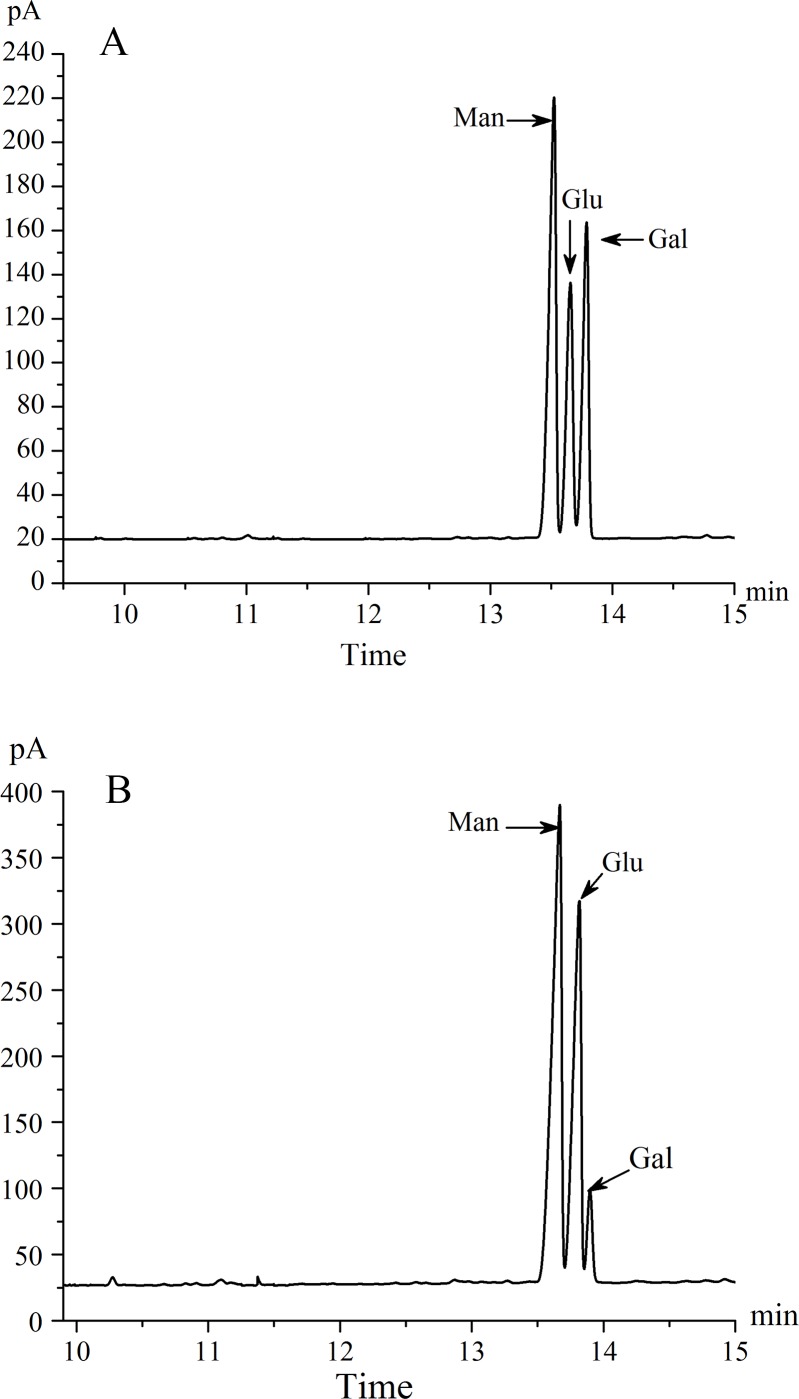
(A) GC chromatogram showing the monosaccharide composition of UP2; (B) GC chromatogram showing the monosaccharide composition of UP2-0.5M.

The monosaccharide composition of UP2 was analyzed by GC (**Fig 3A**), revealing that UP2 is composed mainly of mannose, glucose, and galactose at a molar ratio of 1.7:1.0:1.2.

Partial hydrolysis analysis was performed to obtain more information on the structure of UP2. The GC chromatogram of the fraction inside the dialysis bag (UP2-0.5M) compared with those of the monosaccharidic standards glucose, galactose, rhamnose, mannose, xylose, and arabinose was shown in **Fig 3B**, which suggested that UP2-0.5M contained mainly mannose, glucose, and galactose. However, the relative content of galactose was decreased compared to the composition indicated in **Fig 3A**, indicating that the galactose is located on the branch.

In order to obtain information on the glycosidic linkage types present in UP2, it was methylated by the Hakomori method [38] and analyzed by GC-MS. The results showed the absence of peak at 3410 cm^-1^ from the FT-IR spectrum of methylated UP2 (**Fig 2B**) which suggested that the methylation was completed and the absorption peak at 2925 cm^-1^ for the C-H stretching vibrations in methylated UP2 was obviously enhanced by the addition of a methyl group [8, 34]. The methylated UP2 was hydrolyzed, reduced, acetylated, and converted into methylated alditol acetates that were analyzed by GC-MS (**Table 1**). Five partially methylated alditol acetates, i.e., 1, 5-di-acetyl-2,3,4,6-tetra-*O*-methyl galactitol, 1,5,6-tri-acetyl-2,3,4-tri-*O*-methyl glucitol, 1,5,6-tri-acetyl-2,3,4-tri-*O*-methyl galactitol, 1,5,6-tri-acetyl-2,3,4-tri-*O*-methyl mannitol, and 1,3,5,6-tetra-acetyl-2,4-di-*O*-methyl glucitol were detected. Correspondingly, the following five glucosidic linkages (1 → 6)-linked glucosyl (residue A), (1 → 3,6)-linked glucosyl (residue B), non-reducing terminal galactosyl (residue C), (1 → 6)-linked galactosyl (residue D), and (1 → 6)-linked mannosyl (residue E) were present in UP2 at a molar ratio of 0.7:4.6:4.1:2.2:9.1. Furthemore, the methylation analysis indicated that UP2 was a branched heteropolysaccharide.

**Table 1:** GC-MS date for methylation analysis of UP2.

Methylated sugars	Linkages types	Molar ratios	Mass fragments (m/z)
2,3,4,6-Me_4_-Gal*p*	Terminal	4.1	45,71,87,101, 117,129,145,161,205
2,3,4-Me_3_-Glc*p*	1,6-Linked- Glc*p*	0.7	45,59,71,87,99,101,117,129,161, 189
2,3,4-Me_3_-Gal*p*	1,6-Linked- Gal*p*	2.2	45,59,71,87,101,117,129, 161,189,233
2,3,4-Me_3_-Man*p*	1, 6-Linked- Man*p*	9.1	45, 59,71,89, 117,129, 161, 189,233
2, 4-Me_2_-Glc*p*	1,3,6-Linked- Glc*p*	4.6	45, 59,71,87,99, 103,117,129, 189

**Fig 4:**
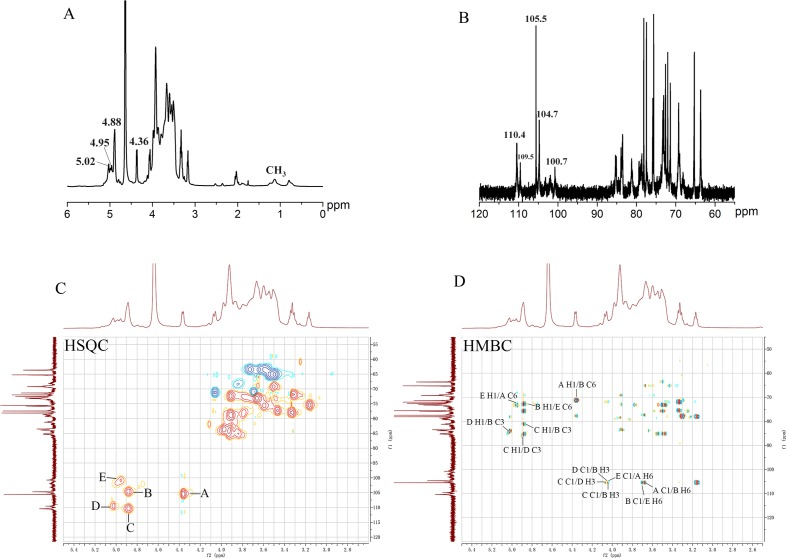
(A) ^1^H NMR spectrum of UP2 in D_2_O; (B) ^13^C NMR spectrum of UP2 in D_2_O; (C) HSQC NMR spectrum of UP2 in D_2_O; (D) HMBC NMR spectrum of UP2 in D_2_O.

UP2 was further subjected to NMR analysis to determine its structural features. All ^1^H and ^13^C NMR signals were assigned to UP2 using 2D NMR spectra and reference data [27, 32, 39]. The NMR spectra of UP2 were shown in **Fig 4**. According to previous studies [2, 4], the anomeric hydrogen chemical shifts of UP2 appear at *δ* 5.02, 4.95, 4.88 and 4.36 ppm. The heteronuclear single-quantum coherence (HSQC) spectrum of UP2 (**Fig 4C**), showed overlapping peaks around *δ* 4.88 ppm, indicating the presence of two different connections. ^1^H NMR is an effective way to determine whether sugar residues are present in the *α*- or *β*-configuration [39]. The chemical shifts at *δ* 5.03 and *δ* 4.96 ppm were ascribed to the α-pyranose configuration. The others were assigned to the *β*-pyranose configuration. The signals at *δ* 4.36, 4.88, 4.88, 5.02, and 4.95 ppm in **Fig 4A** were assigned to the anomeric protons of the sugar residues A–E. The solvent proton peak at δ 4.64 ppm was used as a reference for the absorption peaks, and the chemical shifts from *δ* 4.18 to δ 3.06 ppm correspond to the absorption of H_2_-H_6_. The signals at ca. *δ* 1.23 and *δ* 2.09 ppm in **Fig 4A** were assigned to the CH_3_ and acetyl groups in UP2 [27, 39].

The ^13^C NMR spectrum of UP2 was shown in **Fig 4B**. Five signals in the anomeric region from *δ* 95–115 ppm were determined. The anomeric carbon signal peaks at *δ* 110.4, *δ* 109.5, *δ* 105.5, *δ*104.7, and *δ* 100.7 ppm correspond to C-1 of the non-reducing terminal *β*-*D*-galactosyl residues, (1 → 6)-linked *α*-*D*-galactosyl residues, (1 → 6)-linked *β*-*D*-glucosyl residues, (1 → 3, 6)-linked *β*-*D*-glucosyl residues, and (1 → 6)-linked *α*-*D*-mannosyl residues, respectively. The correlation of the anomeric carbon signals with their respective protons was revealed in the HSQC spectrum (**Fig 4C**), which showed cross-peaks for *δ* 4.36/105.5 (A), *δ* 4.88/104.7 (B), *δ* 4.88/110.4 (C), *δ* 5.02/109.5 (D), and *δ* 4.95/100.7 (E). The ^1^H and ^13^C chemical shifts for UP2 were listed in **Table 2**.

**Table 2:** Chemical shifts of resonances in the ^1^H NMR and ^13^C NMR spectra of UP2.

Glycosyl residues	H-1/C-1	H-2/C-2	H-3/C-3	H-4/C-4	H-5/C-5	H-6/C-6
**A** →6)-*β-D*-Glc*p*-(1→	4.36/105.5	3.16/75.4	3.47/77.3	3.46/71.5	3.35/75.9	3.55/72.6
**B** →3,6)-*β-D*-Glc*p*-(1→	4.88/104.7	4.05/71.1	3.84/85.2	3.32/71.8	3.30/71.9	3.84/68.1
**C** *β-D*-Gal*p*-(1→	4.88/110.4	3.32/77.9	3.79/78.2	3.68/82.3	3.93/83.7	3.50/69.2
**D** →6)-*α-D*-Gal*p*-(1→	5.02/109.5	3.89/78.8	3.63/79.1	3.97/83.6	4.11/81.3	3.92/72.7
**E** →6)-*α-D*-Man*p*-(1→	4.95/100.7	3.91/72.4	3.66/73.0	3.65/69.1	3.59/75.6	3.69/71.2

The heteronuclear multiple-bond correlation (HMBC) spectrum indicates the connectivity of the sugar residues. The anomeric carbon signal of residue **A** at δ 105.5 was confirmed by the presence of cross-peaks for **A** H-1, **E** C-6 and **E** H-6, **A** C-1 (**Fig 4D**). The inter-residue HMBC correlations from H-1 of residue **A** to C-6 of residue **E** and H-6 of residue **E** to C-1 of residue **A** indicated the linkage of C-1 of residue **A** to the *O*-6 position of residue **E**. The anomeric carbon signal of residue **B** at δ 104.7 was confirmed by the cross-peaks **B** H-1, **A** C-6 and **A** H-6, **B** C-1 in the HMBC experiment (**Fig 4D**). The inter-residue HMBC correlations from H-1 of residue **B** to C-6 of residue **A** and H-6 of residue **A** to C-1 of residue **B** indicated the linkage of C-1 of residue **B** to the *O*-6 position of residue **A**. The ^13^C chemical shifts for residue **C** at δ 110.4 could be assigned to the *D*-galactosyl residue, which was confirmed by the coupling corresponding to **C** C-1, **D** H-6 and **C** C-1, **B** H-3 detected in the HMBC experiment (**Fig 4D**), and revealed that the terminal residue **C** was attached at C-6 of residue **D** or situated at C-3 of residue **B**. The ^13^C signal of residue **D** at δ 109.5 was confirmed by the cross-peaks **D** H-1, **B** C-3 and **B** H-3, **D** C-1 in the HMBC experiment (**Fig 4D**). The inter-residue HMBC correlations from H-1 of residue **D** to C-3 of residue **B** and H-3 of residue **B** to C-1 of residue **D** indicated the branch substitution situated at C-3 of residue **B.** Furthermore, the linkage of residues C-1 of residue **D** to the *O*-3 position of residue **B** was indicated. The anomeric carbon chemical shift for residue **E** at δ 100.7 was confirmed by the cross-peak **E** H-1, **B** C-6 in the HMBC experiment (**Fig 4D**), indicated that residue **E** was attached at C-6 of residue **B**. These results indicated that UP2 mainly consisted of a backbone chain comprising (1 → 6)-*β*-*D*-Glc*p*, (1 → 3, 6)-*β*-*D*-Glc*p* and (1 → 6)-*α*-*D*-Man*p* linkages, and the branch chains were (1 → 6)-*α*-*D*-Gal*p* and *β*-1-*D*-Gal*p*.

**Fig 5:**
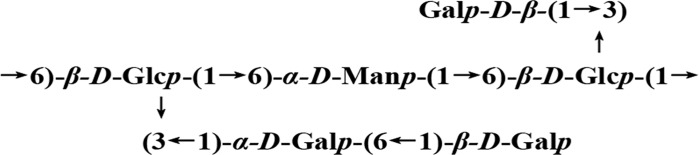
Predicted structure of the repeating unit of UP2.

Based on the monosaccharide composition analysis, methylation analysis, and NMR analysis, it may be deduced that UP2 was a heteroglucan consisting of a (1 → 6)-linked *β*-*D*-Glc*p* and (1 → 6)-linked *α*-*D*-Man*p* backbone with intermittent 1-linked *β*-*D*-Gal*p* or (1 → 6)-*α*-*D*-Gal*p* branched at the *O*-3 position of (1 → 6)-linked *β*-*D*-Glc*p*. Thus, the possible repeating structural unit of UP2 has been suggested in **Fig 5**.

### Immunological activity analysis

**Fig 6:**
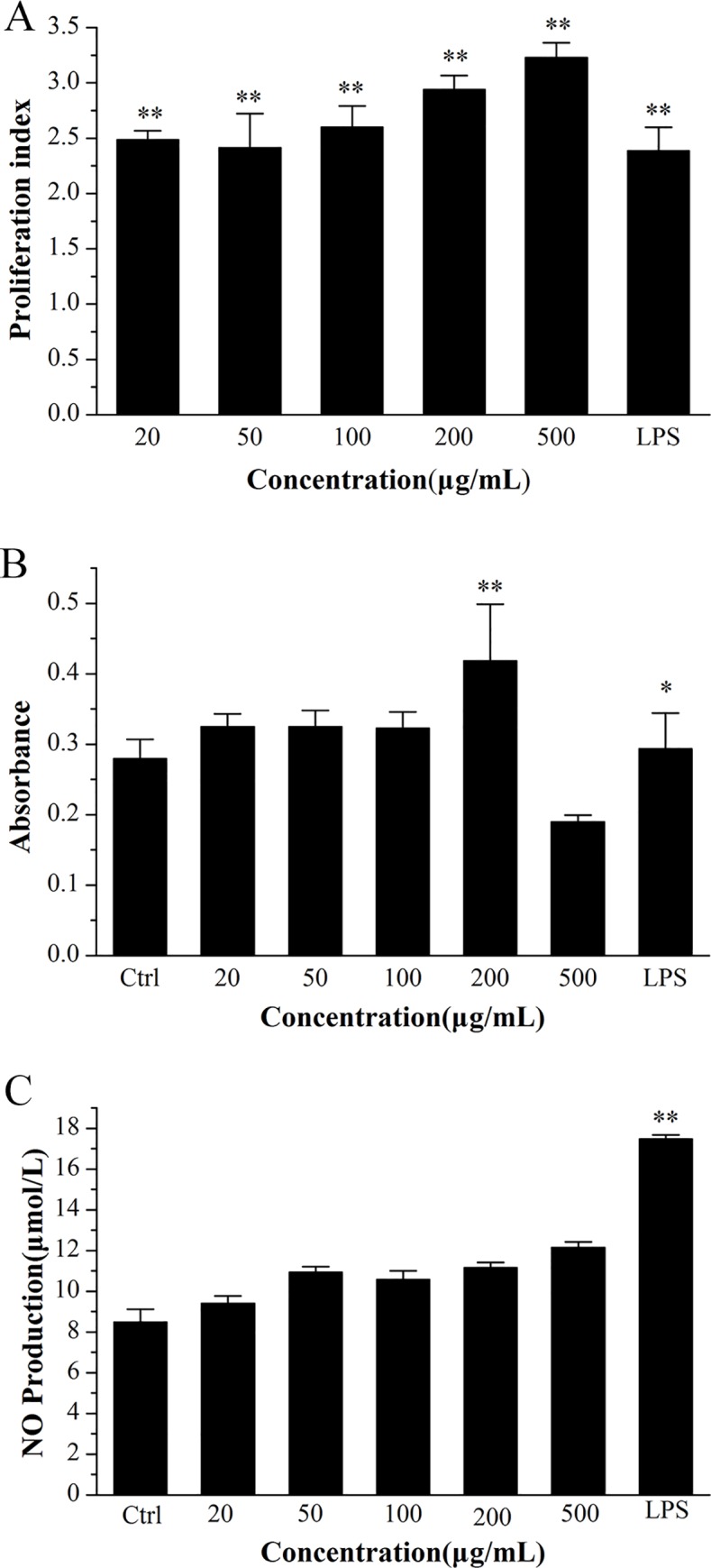
**Effect of UP2 on the cell proliferation (A), phagocytosis activity (B), and NO production (C) of RAW264.7 macrophages.** All experiments were repeated at least three times. The data values are expressed as mean ± SD (n ≥ 3). Significant difference: *P < 0.05, and **P < 0.01 for difference from the control without treatment.

The immune system is an important system of the human body to perform an immune response and immune function [39]. It maintains the stability of the human body, eliminates the antigenic substances and plays an immunological surveillance role in the human body [1, 6, 14–15]. It is reported that some natural polysaccharides isolated from different organs displayed a variety of biological effects via activating macrophage immune system functions [3–4, 32]. Macrophages play an important role in the mammalian immune system, in that they not only initiate innate immunity, but also regulate adaptive immunity [40]. Commonly, murine macrophage cell line RAW264.7 cells are used to investigate immunostimulatory activity [41]. In our previous studies, the polysaccharide from *U*. *esculenta* (HSSE) was shown to possess immunomodulatory potential [1]. Consequently, the immunostimulatory activity of UP2 upon RAW264.7 macrophages was assessed by determining their cell proliferation, phagocytic activity, and NO production. The effect of UP2 on the proliferation of RAW264.7 cells based on MTT assay was shown in **Fig 6A**. The proliferative effects of UP2 were significant up to a concentration of 500 μg/mL compared with those of the blank control, indicated that the polysaccharide stimulated RAW264.7 cell proliferation. The proliferation index of UP2 increased from 2.4 to 3.2 as the sample concentration increased from 50–500 μg/mL. According to the data reported, UP2 showed higher cells proliferation ability than the protein-bound polysaccharide (GSP-4) in Han’s paper [6]. Du *et al*. reported the polysaccharides [1] showed lower proliferation ability than the UP2 in this study. Furthermore, UP2 exhibited no cytotoxicity.

Macrophages can phagocytose some pathogens *in vivo* or *in vitro* [3]. Therefore, the phagocytic activity of the macrophages was examined by neutral red phagocytosis assay, and the results were shown in **Fig 6B**. Compared with the blank control, the phagocytic activity of RAW264.7 cells was significantly increased by UP2 at a concentration of 200 μg/mL. However, after 48 h treatment with 500 μg/mL of UP2, the phagocytic activity on RAW264.7 cells was lowest. We speculated that this effect was due to the resistance of RAW264.7 cells being induced at high concentration.

NO is one of the cell factors that plays a significant role in the immune response [1, 3]. Hence, we investigated the effect of UP2 on the NO production of macrophages by the Griess method, and the results were shown in (**Fig 6C**). Compared with the blank control, treatment of RAW264.7 cells with LPS causes a remarkable increase in NO release. At concentration of 500 μg/mL, the NO production was 45% higher than that of the blank control. The level of NO production under a UP2 concentration of 200 μg/mL reached 11.2 μmol/L. However, at a low UP2 concentration (20 μg/mL), the NO production of RAW264.7 cells was not obvious, but was still 12% higher than the blank control. These results indicated that UP2 stimulated the production of NO from RAW264.7 cells.

Macrophages play a central role in the immune system, combating infection and inflammation by phagocytosis and the secretion of inflammatory factors such as NO [40, 42]. When the macrophages were exposed to the UP2, the cells exhibited the ability to stimulate phagocytosis and release NO. Thus, it is suggested that UP2 may be used as a potential immunomodulatory agent. Moreover, *Umbilicaria esculenta* was a beneficial material and UP2 was also a novel polysaccharide, as well as UP2 showed a remarkable biological effect. Polysaccharides have been found to exert remarkable effects on the immune system. Bi *et al*. among others have reported that the polysaccharide from *Bulgaria inquinans* with (1 → 6)-*β*-*D*-Glc*p* linkages was a novel immune stimulant [43]. Since then, several polysaccharides extracted from fungi and lichens have been shown to exhibit antitumor and immunostimulatory activity.

## Conclusion

In the present study, a novel water-soluble polysaccharide, which we termed UP2, was isolated from *U*. *esculenta* cultivated in the Huangshan Mountain and further purified by anion-exchange chromatography on a column of DEAE-cellulose to obtain a homogeneous polysaccharide. UP2 was shown to be composed mainly of mannose, glucose, and galactose at a molar ratio of 1.7:1.0:1.2, with an average molecular weight of 3.33 × 10^5^ Da. GC-MS analysis revealed that the linkages in UP2 were (1 → 6)-linked Glc*p*, (1 → 3,6)-linked Glc*p*, *t*-linked Gal*p*, (1 → 6)-linked Gal*p*, and (1 → 6)-linked Man*p* at a molar ratio of 0.7:4.6:4.1:2.2:9.1. The backbone of UP2 was shown to consist of (1 → 6)-linked *β-D*-Glc*p* and (1 → 6)-linked *α*-*D*-Man*p* with (1 → 6)-linked *α*-*D*-Gal*p* or 1-linked *β*-*D*-Gal*p* branches occasionally substituted at the *O*-3 position of (1 → 6)-linked *β*-*D*-Glc*p*. UP2 stimulated the proliferation of RAW264.7 cells, as well as enhancing their phagocytosis and increasing their NO production. Consequently, it is suggested that UP2 may be used as a novel immune-modulatory agent.

## Supporting Information

S1 Dataset Elution profiles of crude UP. (XLSX)

S2 Dataset HPGPC chromatogram of UP2. (XLSX)

S3 Dataset FT-IR spectrum of UP2. (XLSX)

S4 Dataset FT-IR spectrum of methylated polysaccharide. (XLSX)

S5 Dataset GC chromatogram of monosaccharides composition of UP2. (XLSX)

S6 Dataset GC chromatogram of monosaccharides composition of UP2-0.5M. (XLSX)

S7 Dataset GC-MS data for methylation analysis of UP2. (XLSX)

S8 Dataset cell proliferation. (XLS)

S9 Dataset phagocytosis activity. (XLS)

S10 Dataset NO production. (XLS)

S1 Fig ^1^H NMR spectrum of UP2. (PDF)

S2 Fig ^13^C NMR spectrum of UP2. (PDF)

S3 Fig HSQC NMR spectrum of UP2. (PDF)

S4 Fig HMBC NMR spectrum of UP2. (PDF)
