# Supplementary figures and images for: Structure Elucidation of a Polysaccharide from Umbilicaria esculenta and Its Immunostimulatory Activity
Source: PLoS One. 2016 Dec 20;11(12):e0168472. doi: 10.1371/journal.pone.0168472 (PMC5172621; doi:10.1371/journal.pone.0168472)

PROTON\_01  
wjh-xjl-3

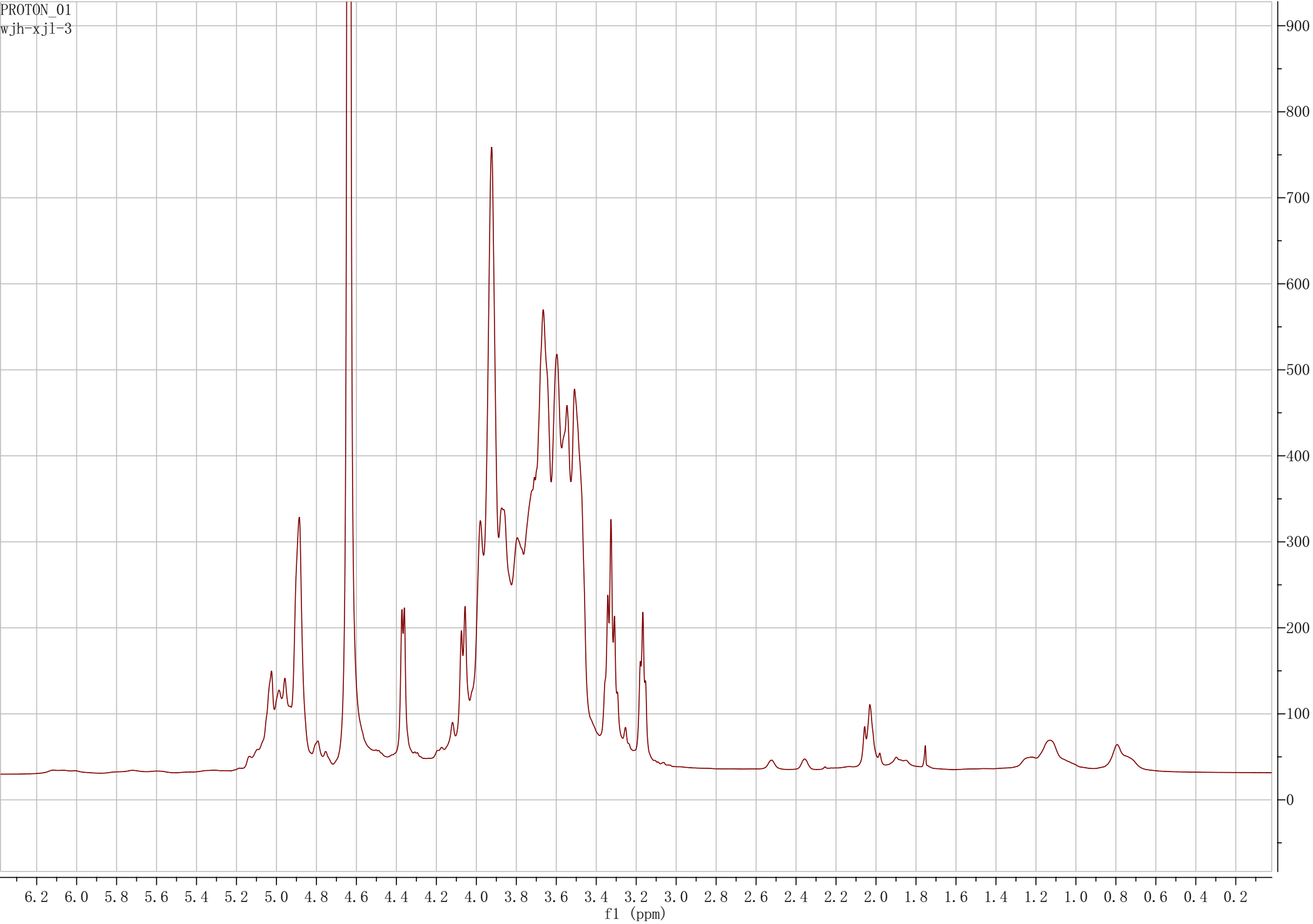

Supplement: S1 Fig — (PDF) [file pone.0168472.s011.pdf]

CARBON\_01  
wjh-xj1-3

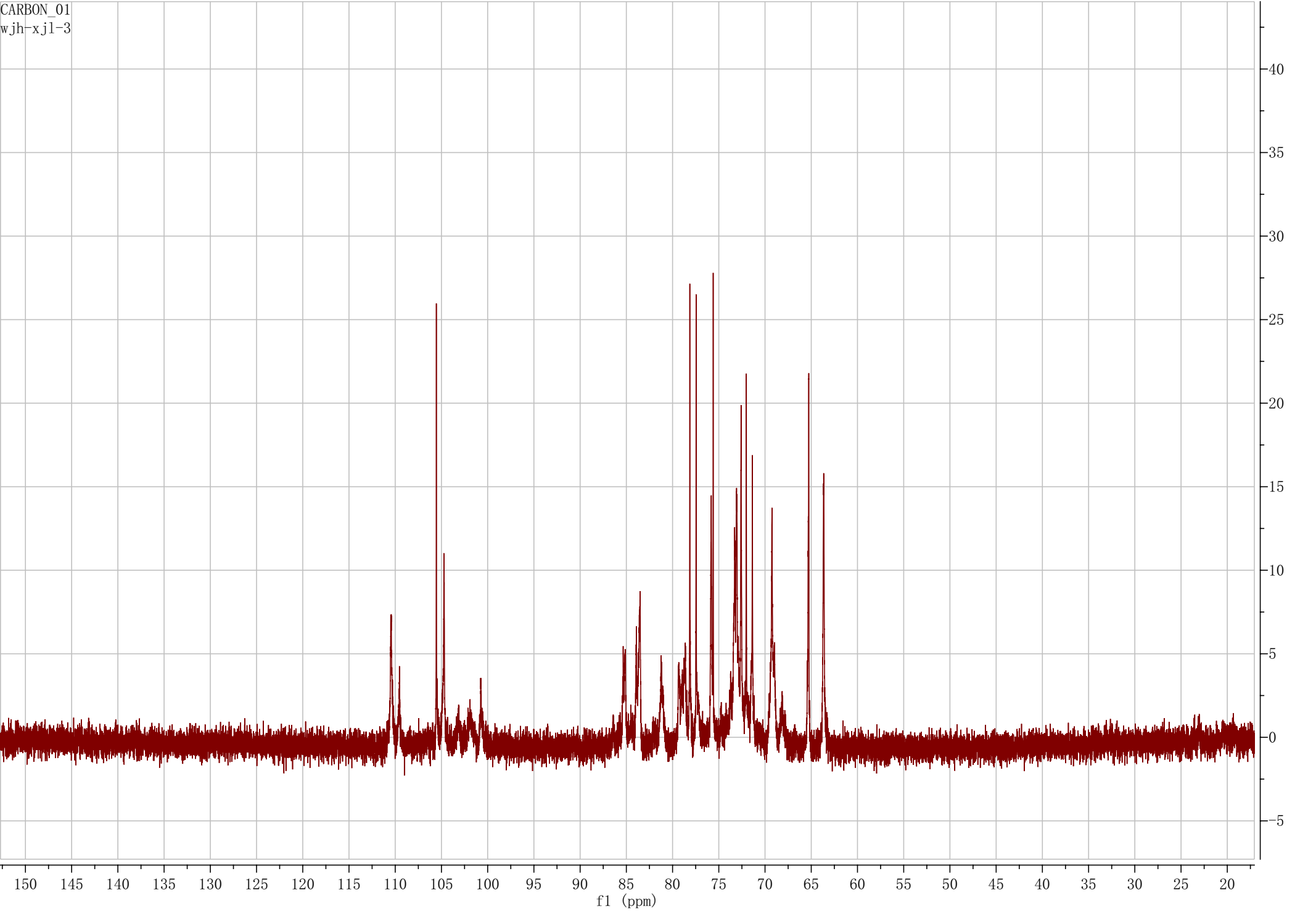

Supplement: S2 Fig — (PDF) [file pone.0168472.s012.pdf]

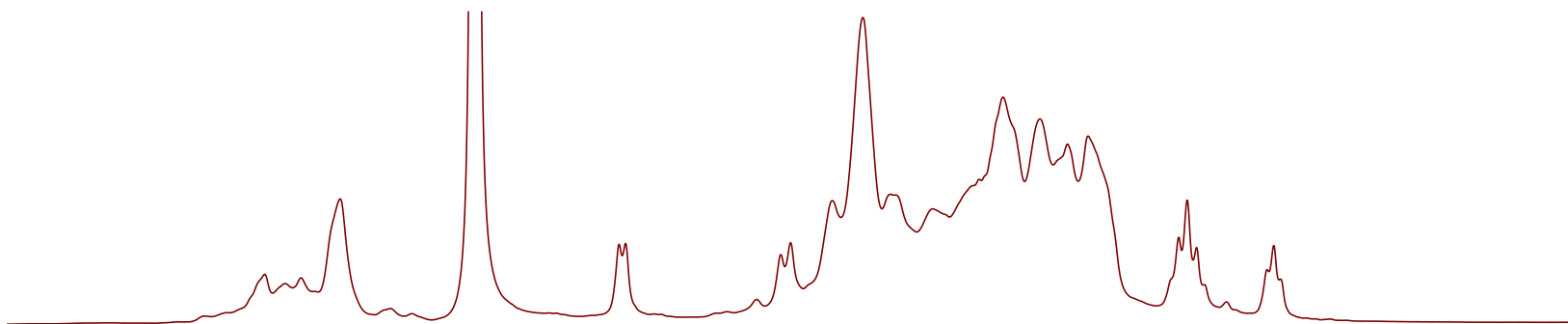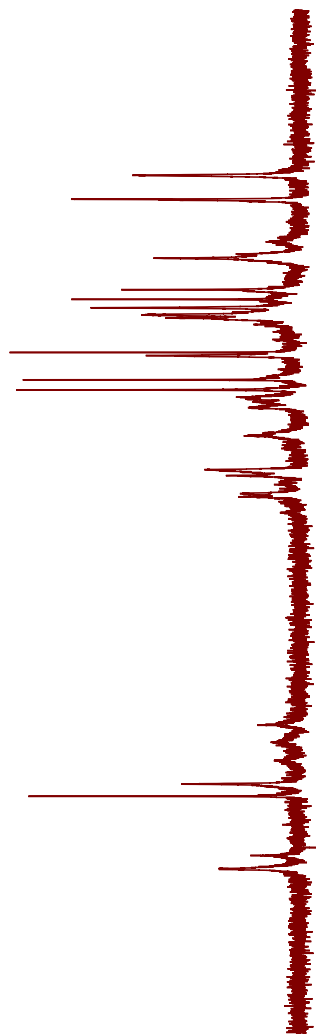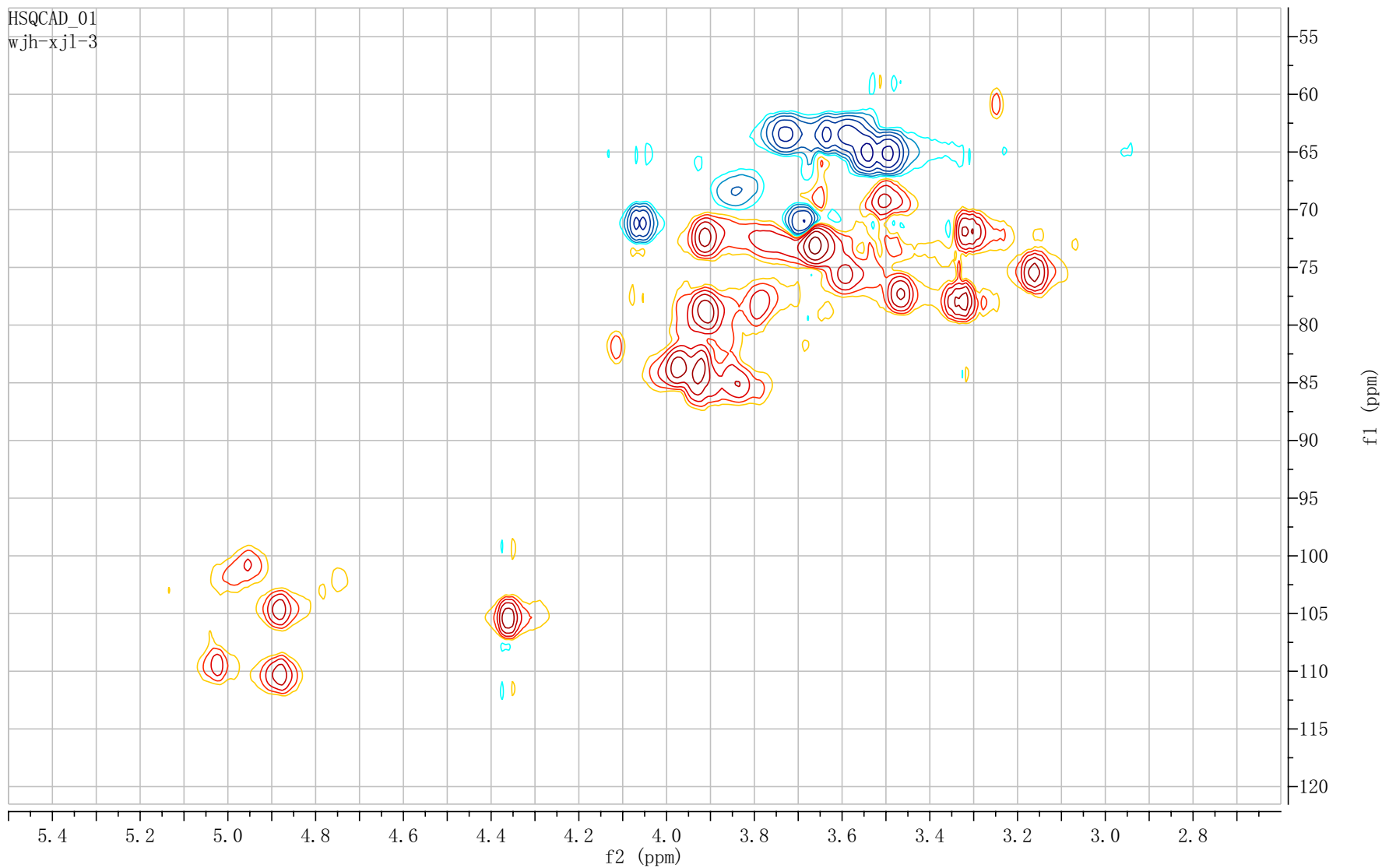

Supplement: S3 Fig — (PDF) [file pone.0168472.s013.pdf]

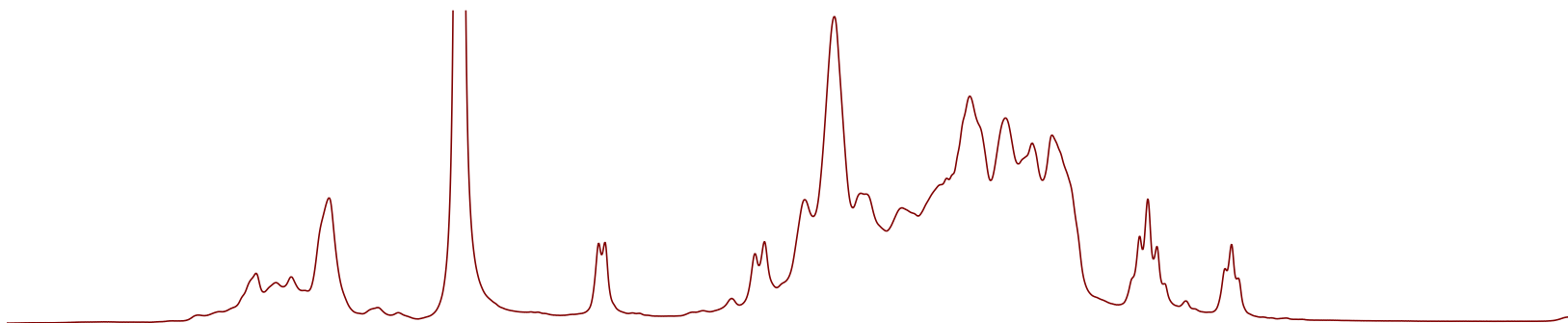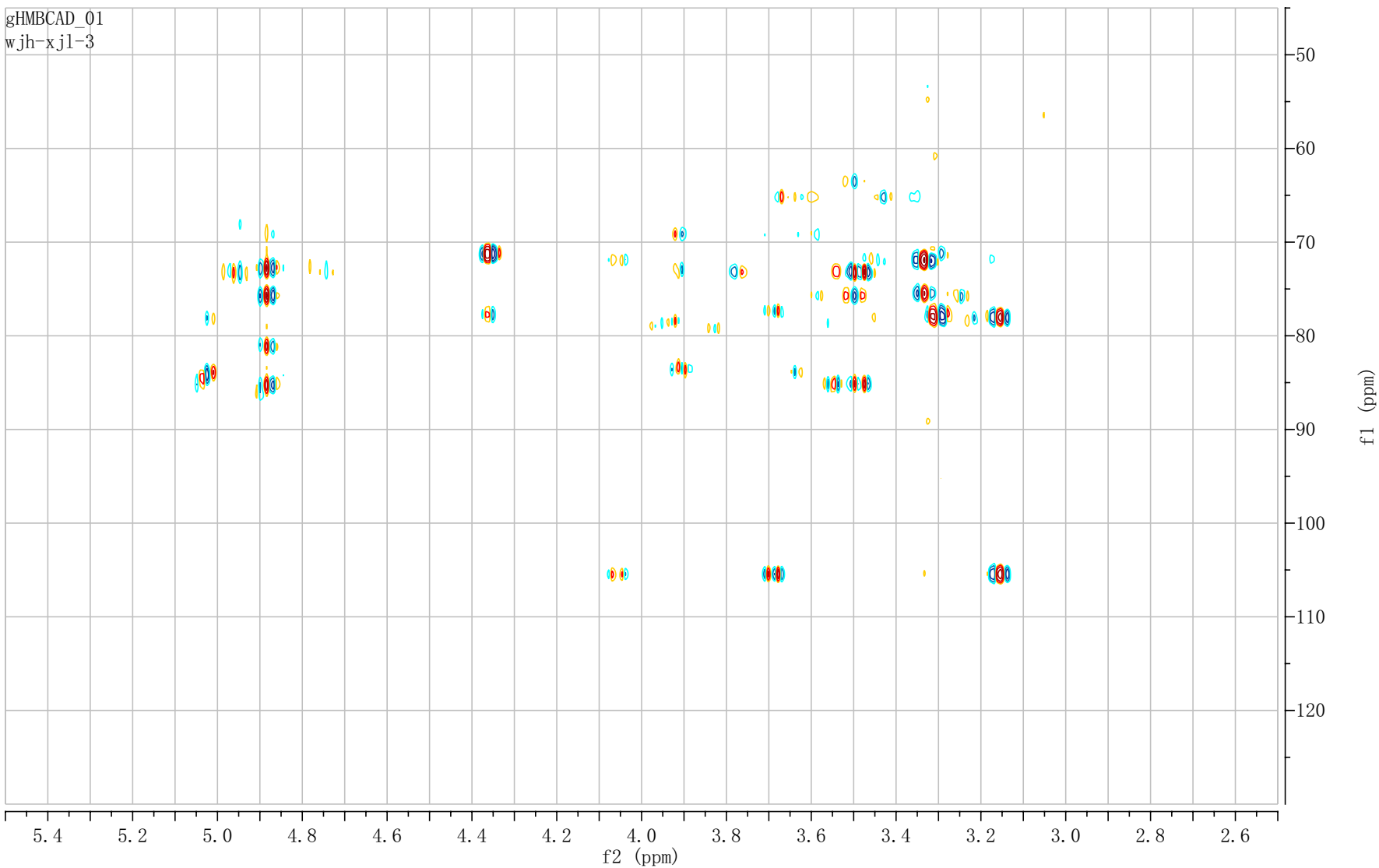

Supplement: S4 Fig — (PDF) [file pone.0168472.s014.pdf]
